# Mohs micrographic surgery for treatment of non–melanoma skin cancer involving the nipple-areola complex: A case series of 8 patients

**DOI:** 10.1016/j.jdcr.2023.09.029

**Published:** 2023-10-10

**Authors:** Somaira Nowsheen, Bobak Pousti, Adam Miller, Shaundra Eichstadt, Shang I. Brian Jiang

**Affiliations:** Department of Dermatology, University of California San Diego, San Diego, California

**Keywords:** areola, breast cancer, case series, dermatology, Mohs, nipple, retrospective review, skin cancer, surgery

## Introduction

Skin cancer is the most frequently diagnosed malignancy, with non–melanoma skin cancers (NMSCs)—basal cell carcinoma (BCC) and squamous cell carcinoma (SCC)—being the most common.^1^^,^^2^ UV exposure, tumor depth, and immunosuppression are major risk factors for NMSC, with chronic sun exposure being the strongest environmental risk factor.^3^, ^4^, ^5^ Although rare, cutaneous malignancy can involve the nipple or areola, and potentially, these tumors can be more aggressive and require specialized care.^6^, ^7^, ^8^ There are limited data available on the epidemiology, risk factors, and optimal management of this condition. Herein, we report the characteristics and surgical outcomes of patients diagnosed with cutaneous malignancy involving the nipple-areola complex (NAC) treated with Mohs micrographic surgery (MMS) at our institution.

For this case series, a retrospective chart review of patients at the University of California San Diego (UCSD) Dermatologic Surgery Unit with NMSC on the NAC between January 1, 2007 and January 1, 2022, was conducted, and 8 patients were identified. Patient and tumor characteristics, surgical details, and adverse outcomes were noted. Adverse outcomes were defined as any complications requiring additional medical or surgical intervention and included skin infections, postoperative bleeding, flap necrosis, graft loss, and wound dehiscence. The surgical margins were determined by taking the difference between the preoperative and postoperative excision dimensions.

## Case series

From January 1, 2007 to January 1, 2022, 8 patients—7 males and 1 female—underwent surgical interventions at the Department of Dermatology at UCSD for NMSC on the NAC (Table I). The mean age at diagnosis was 70.7 ± 14.5 years. Six patients (75%) had BCC, and 2 patients (25%) had SCC on the NAC. All 8 patients underwent MMS for the excision of cutaneous malignancy involving the NAC. The average preoperative size of the skin cancers was 12.1 ± 4.2 mm (range: 5-20 mm), and the average postoperative size was 25.1 ± 10.3 mm (range: 12-47 mm). The final surgical margin was 13.0 ± 9.8 mm (range: 7-36 mm), and the final surgical length was 5.2 ± 17 cm (range: 3.4-8 cm) for linear closures and 1.9 ± 1.1 cm^2^ (range: 0.5-2.9 cm^2^) for flaps. On average, 2 stages of MMS were needed for histologically clear margins, with a range of 1 to 4 stages. No complications or recurrences were noted over a mean follow-up of approximately 4 years with dermatology.

**Table I:** Characteristics and surgical outcomes of 8 patients diagnosed with cutaneous malignancy involving the nipple or areola at a single institution∗

Variable	Case 1	Case 2	Case 3	Case 4	Case 5	Case 6	Case 7	Case 8
Age at diagnosis (years)/sex	67/M	44/M	84/M	90/M	66/M	74/M	59/M	75/F
No. of prior NMSC (*n*)	5	0	8	15	12	5	1	0
History of surgery or radiation on the chest/breast	No	No	No	No	No	No	No	Yes (radiation and surgery)
History of immunosuppression	No	No	No	No	No	No	No	Yes†
History of solid organ malignancy	No	No	No	No	No	No	No	Yes
History of autoimmune disease	No	No	No	Yes (diabetes)	No	No	No	Yes (thyroid disease)
Location of cancer	Areola	Areola	Areola	Areola + nipple	Areola	Nipple	Areola + nipple	Areola
Type of skin cancer on the breast	Nodular BCC	Nodular BCC	SCC	SCC	Multicentric BCC	Nodular BCC	Nodular BCC	Nodular BCC
Type of surgery	MMS	MMS	MMS	MMS	MMS	MMS	MMS	MMS
Preoperative size (mm)‡	10	11	13	13	14	20	11	5
Postoperative size (mm)§	20	27	25	20	21	29	47	12
Final surgical margin (mm)	10	16	12	7	7	9	36	7
Final surgical length and type of repair (cm)	3.4 cm (CLC)	4.7 cm (CLC)	6.2 cm (CLC)	3.6 cm (CLC)	2.5 cm^2^ (advancement flap)	1.5 cm^2^ (C-V transposition flap), 5 cm (CLC)	0.5 cm^2^ (C-V transposition flap), 8 cm (CLC)	2.9 cm^2^ (advancement flap)
Stages needed for histologically clear margin (n)	3	2	2	1	2	2	4	2
Repair material	Sutures	Sutures	Sutures	Sutures	Sutures	Sutures	Sutures	Sutures
Any complications observed	No	No	No	No	No	No	No	No
Recurrence noted	No	No	No	No	No	No	No	No
Follow-up with dermatology (mo)	81	8	1	24	129	1	36	5
Follow-up at UCSD (mo)	84	8	13	27	129	1	45	17

*BCC*, Basal cell carcinoma; *CLC*, complex linear closure; *MMS*, Mohs micrographic surgery; *NMSC*, non–melanoma skin cancer; *SCC*, squamous cell carcinoma; *UCSD*, University of California San Diego.

∗ Each patient had 1 unique skin cancer on the breast. No patient had a history of melanoma, transplant, or local trauma to the breast.

† Patient was previously on chemotherapy for treatment of triple-negative invasive ductal carcinoma of the breast (adriamycin/cytoxan, followed by dose-dense taxol and radiation)

‡ Greater of the 2 dimensions of the lesion.

§ Greater of the 2 dimensions of the defect.

## Discussion

Cutaneous malignancies involving the NAC may require a multidisciplinary approach, and MMS should be considered as the first-line treatment for these cases. MMS is a surgical technique used to remove skin cancer that spares normal tissue with high cure rate. It is particularly useful for treating skin cancer in areas where tissue sparing is important functionally and/or cosmetically and in areas that are difficult to reconstruct, such as the NAC.

A review of the literature on recurrence rates of NMSC in terms of treatment type revealed 113 previously reported cases of patients diagnosed with BCC on the NAC (Table II). Of these patients, 6 experienced a recurrence when treated with topical 5-fluorouracil (5-FU, *n* = 2), excision (*n* = 3), or a combination of excision and radiation therapy (*n* = 1), resulting in an overall recurrence rate of 7.9% (6/76). However, the recurrence rate with MMS was 0% (0/17), which is consistent with that in our report (0/6). The recurrence rate with excision in the literature was 5.9% (3/51). Interestingly, 2 patients with superficial BCC underwent treatment with topical 5-FU and both had a recurrence (100%, 2/2), suggesting that 5-FU topical therapy may not have the same efficacy for the treatment of superficial BCC at this site. No patient underwent excision or topical therapy for NMSC of the NAC in our cohort. There are 11 previously reported cases of SCC on the NAC; none of these patients experienced a recurrence with surgical intervention, 1 of which included MMS. This is also in agreement with our report (0/2 recurrence with MMS). However, of the 8 reported cases of SCC in situ on the NAC, a 50% (2/4) recurrence rate was noted after standard excision. One patient treated with photodynamic therapy (1/1) also experienced recurrence of malignancy. None of the patients in our cohort had SCC in situ of the NAC.

**Table II:** Recurrence rates of NMSC of NAC by treatment type, as reported in the literature

Type of NMSC	Total No. of patients	No. of patients with recurrence	Overall recurrence rate	Recurrence rate with MMS	Recurrence rate with excision	Recurrence rate with topical therapy∗
BCC	113	6	7.9% (6/76)	0% (0/17)‡	5.9% (3/51)	100% (2/2)
SCC†	11	0	0% (0/9)§	0% (0/1)	0% (0/3)‖	–
SCC in situ	8	3	50.0% (3/6)§	–	50.0% (2/4)§	–

*BCC*, Basal cell carcinoma; *MMS*, Mohs micrographic surgery; *NAC*, nipple-areola complex; *NMSC*, non–melanoma skin cancer; *SCC*, squamous cell carcinoma.

∗ Includes 5-fluorouracil.

† Includes 1 patient with basosquamous cell carcinoma.

‡ Data missing for 3 patients.

§ Data missing for 2 patients.

‖ Data missing for 1 patient.

**Fig 1:**
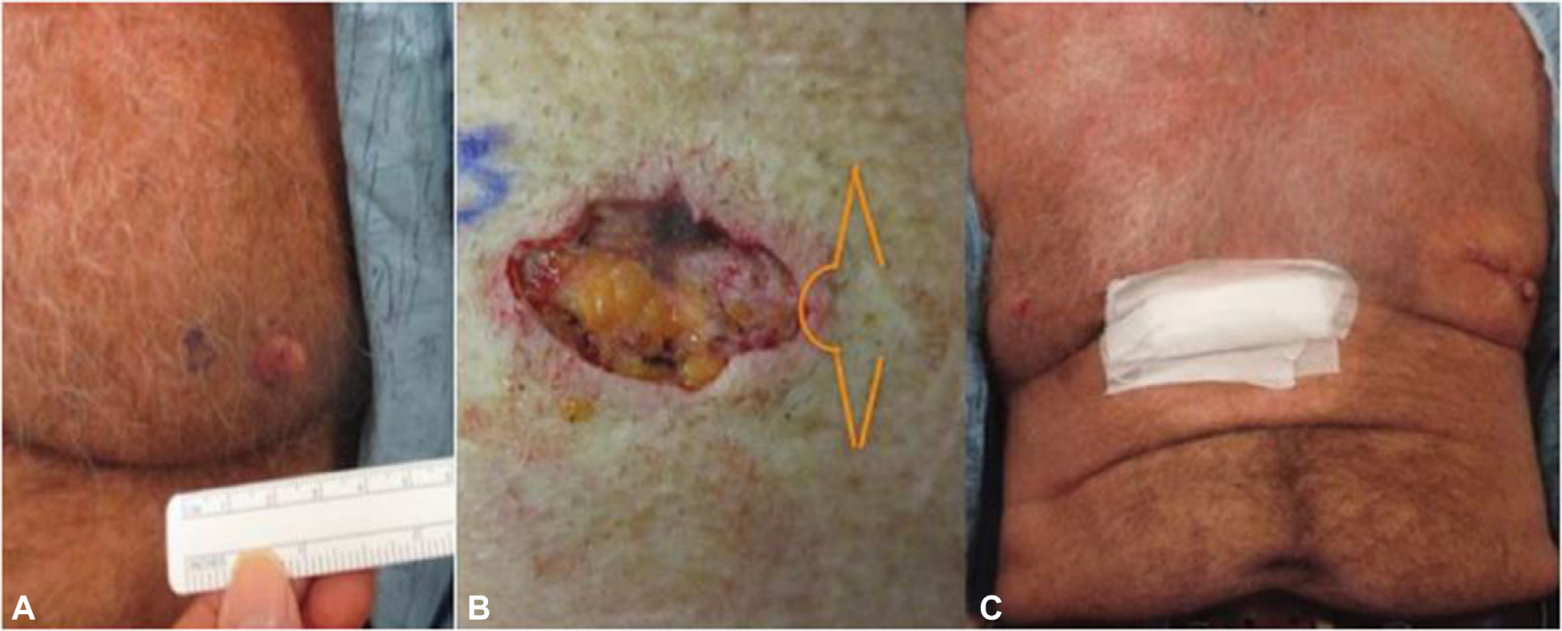
**A,** Preoperative photograph of BCC of the NAC. **B,** Postoperative defect with no remaining residual tissue of the NAC and C-V flap design in orange. **C,** Postreconstruction of the left side of the nipple after MMS showing reasonable symmetry in location and projection of the reconstructed nipple to the contralateral nipple. *MMS*, Mohs micrographic surgery; *NAC*, nipple-areolar complex. Represented with permission from Dermatologic Surgery.

We had previously reported on the use of C-V transposition flaps for defects on the NAC after MMS (Fig 1).^9^ This approach, which is commonly used in the plastic surgery field for repair after mastectomy, contributes to improved aesthetic outcomes and patient satisfaction by addressing the challenges of NAC reconstruction after MMS, which can be particularly demanding owing to the need for both structural and cosmetic restoration.

A review of available data of cases of BCC treated with MMS in the literature revealed a mean preoperative size of 17.3 mm, and postoperative size of 25.3 mm, and a final surgical margin of 8 mm achieved over an average of 2 stages (Table III). Notably, detailed information was available for only 6 of the 113 cases of BCC. Herein, we contribute 6 cases of BCC of the NAC and 2 cases of SCC of the NAC to the literature, of which there is only 1 prior report. This will lead to a significant increase in the number of reported cases and hopefully help to improve our understanding of these rare conditions.

**Table III:** Surgical details of BCC of NAC treated with MMS, as reported in the literature

Variable	Age (y)/Sex	No. of stages (n)	Preoperative size (mm)	Postoperative size (mm)	Final surgical margin (mm)
Case 1	69/Male	4	14	24	10
Case 2	42/Male	1	33	36	3
Case 3	61/Male	1	6	18	12
Case 4	47/Female	2	11	16	5
Case 5	49/Female	3	15	28	13
Case 6	54/Male	1	25	30	5
Mean ± SD	53.7 ± 9.9	2.0 ± 1.3	17.3 ± 9.9	25.3 ± 7.6	8.0 ± 4.2

*BCC*, Basal cell carcinoma; *MMS*, Mohs micrographic surgery; *NAC*, nipple-areola complex

Overall, these findings suggest that MMS is more effective than standard excision in reducing the risk of recurrence of BCC. However, it is important to note that these results are based on a relatively small sample size over a large time frame and that treatments were performed by multiple surgeons at different institutions. Thus, treatment approaches may have varied, and these results may not be generalizable to all patients with NMSC. In addition, given the retrospective nature of the literature review, detailed information regarding surgical margins for the standard excisions were not clearly specified.

Future research should focus on larger sample sizes and incorporate a more comprehensive evaluation of treatment outcomes, including cosmetic outcomes and quality of life, and should aim to better understand the epidemiology, risk factors, and optimal management of this condition.

We conclude that cutaneous malignancy involving the NAC is a rare occurrence, and this case series presented 8 cases of this condition treated with MMS. The review of the literature showed that MMS was a successful surgical treatment for all patients. The final surgical margin was satisfactory, with histologically clear margins achieved after an average of 2 stages, which is in agreement with previously reported cases (Table III). This study also highlighted the comorbidities and skin cancer characteristics of these patients, which can assist dermatologic surgeons in making treatment decisions.

## Conflicts of interest

None disclosed.
